# Wood’s Library for 1884

**Published:** 1884-07

**Authors:** 


WOOD'S LIBRARY FOR 1884.—The efforts of the firm of Wm. Wood & Co., of New York, to give the profession standard medical works at a merely nominal subscription price is commendable, and the profession everywhere should show their appreciation of these efforts by subscribing for the libraries. The first five volumes of the library for 1884 have reached us and compare favorably with those of preceding years. The January volume is Legal Medicine, by Charles Meymott Tidy, M.B., F. C. S., London. It speaks of legitimacy, and paternity, pregnancy, abortion, rape, indecent exposure, sodomy, bestiality, livebirth, infanticide, asphyxia, drowning, hanging, strangulation and suffocation.

It is only necessary to say that this is a work of rare merit and should be in the library of every physician.

The second volume of the library for 1884 is on the Treatment and Pathology of Gonorrhœa, by I. L. Milton, senior surgeon to St. John's Hospital for Diseases of the Skin, London. Fifth Edition. This is a work of 300 pages, in which the author clearly and practically treats gonorrhœa and its complication in every phase. The chapter on pathology is especially interesting. The volume is a valuable addition to the literature of the subject and will well repay perusal.

Diagnosis and Treatment of Diseases of the Heart, by Constantine Paul, member of the Academy of Medicine, Physician to the Lariboisiere Hospital, Paris. Translated from the French.

This volume is profusely illustrated by well executed wood-cuts, which enables the reader to much better understand the various malpositions that this important organ sometimes assumes. The work is well gotten up and contains much that is valuable. The volumes for April and May will be reviewed in the August JOURNAL.

